# Assessing the External Load Associated With High-Intensity Activities Recorded During Official Basketball Games

**DOI:** 10.3389/fpsyg.2021.668194

**Published:** 2021-04-13

**Authors:** Marco Pernigoni, Davide Ferioli, Ramūnas Butautas, Antonio La Torre, Daniele Conte

**Affiliations:** ^1^Department of Biomedical Sciences for Health, University of Milan, Milan, Italy; ^2^Department of Coaching Science, Lithuanian Sports University, Kaunas, Lithuania; ^3^CS Pallacanestro Trapani SSDARL, Trapani, Italy; ^4^IRCCS Istituto Ortopedico Galeazzi, Milan, Italy; ^5^Institute of Sport Science and Innovations, Lithuanian Sports University, Kaunas, Lithuania

**Keywords:** time-motion analysis, physical demands, PlayerLoad, inertial measurement units, accelerometers, microsensors

## Abstract

Load monitoring in basketball is fundamental to develop training programs, maximizing performance while reducing injury risk. However, information regarding the load associated with specific activity patterns during competition is limited. This study aimed at assessing the external load associated with high-intensity activities recorded during official basketball games, with respect to different (1) activity patterns, (2) playing positions, and (3) activities performed with or without ball. Eleven male basketball players (six backcourt, five frontcourt, age: 20.5 ± 1.1 years, stature: 191.5 ± 8.7 cm, body mass: 86.5 ± 11.3 kg; experience: 8.5 ± 2.4 years) competing in the Lithuanian third division were recruited for this study. Three in-season games were assessed *via* time-motion analysis and microsensors. Specifically, the high-intensity activities including sprints, high-intensity specific movements (HSM) and jumps were identified and subsequently the external load [PlayerLoad™ (PL) and PlayerLoad™/min (PL/min)] of each activity was determined. Linear mixed models were used to examine differences in PL, PL/min and mean duration between activity pattern, playing positions and activities performed with or without ball. Results revealed PL was lower in jumps compared to sprints [*p* < 0.001, effect size (ES) = 0.68] and HSMs *(p* < 0.001, ES = 0.58), while PL/min was greater in sprints compared to jumps (*p* = 0.023, ES = 0.22). Jumps displayed shorter duration compared to sprints (*p* < 0.001, ES = 1.10) and HSMs (*p* < 0.001, ES = 0.81), with HSMs lasting longer than sprints (*p* = 0.002, ES = 0.17). Jumps duration was longer in backcourt than frontcourt players (*p* < 0.001, ES = 0.33). When considering activity patterns combined, PL (*p* < 0.001, ES = 0.28) and duration (*p* < 0.001, ES = 0.43) were greater without ball. Regarding HSMs, PL/min was higher with ball (*p* = 0.036, ES = 0.14), while duration was longer without ball (*p* < 0.001, ES = 0.34). The current findings suggest that external load differences in high-intensity activities exist among activity patterns and between activities performed with and without ball, while no differences were found between playing positions. Practitioners should consider these differences when designing training sessions.

## Introduction

Basketball is a popular court-based team sport of intermittent nature, which involves periods of high-intensity activity (e.g., sprinting, jumping) alternated with periods at low- to moderate- intensity (e.g., walking, jogging, running) (Stojanović et al., 2018). Teams implement training strategies with the aim of enhancing players' performance on the court and achieve collective success (Schelling and Torres-Ronda, 2013). In this regard, load monitoring can provide important information to develop more appropriate training programs, maximizing physical performance while preventing overreaching and reducing injury risk (Conte et al., 2018; Ferioli et al., 2020a, 2021). Specifically, training and game load can be determined in terms of internal (e.g., heart rate, hematological markers, Session-Rating of Perceived Exertion) and external (e.g., frequency and duration of activities, distance covered) responses (Fox et al., 2017). Recent research has increasingly focused on quantifying the external demands imposed on basketball players during games (Conte et al., 2020; Ferioli et al., 2020b,c). The assessment of external load in basketball becomes particularly useful when also accounting for the role of contextual factors such as playing position and ball possession status, given that differences have been shown to exist with respect to these variables (Puente et al., 2017; Stojanović et al., 2018; Vázquez-Guerrero et al., 2018, 2019b; Pino-Ortega et al., 2019; Ferioli et al., 2020b; Fernández-Leo et al., 2020; García et al., 2020; Ransdell et al., 2020). Hence, practitioners could use these data to structure training sessions with a clearer understanding of how different activity patterns contribute to the total external load and what differences exist between playing positions and activities which are performed either with or without ball. In particular, high-intensity activities play an important role during competitive basketball games, as players perform quick and forceful movements during key phases of both offensive and defensive possessions (e.g., driving toward the basket, securing a rebound, blocking, screening playing 1-on-1 defense), which are ultimately crucial for team success (Ferioli et al., 2020b). Indeed, the ability to sustain high-intensity activities during competitions is a key component of basketball, as it has been shown to discriminate between players of different competitive levels (Ferioli et al., 2019, 2020c).

Two of the most commonly adopted tools to assess external load in basketball are video-based time-motion analysis (TMA) and wearable inertial measurement units (IMUs) (Fox et al., 2017; Russell et al., 2020). These tools can provide many useful pieces of information including the work:rest ratio and frequency, duration, speed, and distance for specific game activities (Scanlan et al., 2015; Fox et al., 2017; Russell et al., 2020). The extensive use of TMA is justified by its inexpensive nature (Barris and Button, 2008), the capability to provide descriptive information about player activities (Bailey et al., 2017) and the fact that it allows for key elements of performance to be quantified in a valid and consistent manner (Nevill et al., 2008). However, despite the great amount of information that can be obtained, some limitations should be acknowledged. Firstly, the observers' reliability must be systematically assessed to obtain meaningful data. Secondly, data analysis and interpretation is time-consuming, making this method impractical to obtain timely relevant information to adapt the planned training stimulus on a regular basis (Fox et al., 2017). Nonetheless, despite its limitations, TMA is still a valid and reliable method that is widely used in basketball (Conte et al., 2015; Ferioli et al., 2020b,c).

IMUs are a popular alternative to TMA for measuring external load in basketball (Fox et al., 2017) and can provide information about the inertial movements that players execute on the court. Many commercially available IMUs typically integrate a gyroscope, magnetometer and triaxial accelerometer into a single unit (Fox et al., 2017). These devices are able to collect inertial data and combine the instantaneous rate of change of acceleration in all three planes of movement to obtain a single measure of accumulated load, reflective of the external load imposed on the athlete, such as the PlayerLoad™ (PL) and its value per minute played (PL/min) indexes (Catapult Innovation, Melbourne, Australia) (Fox et al., 2017). Furthermore, these devices are usually small and lightweight and can be easily integrated in custom-made straps or vests, which makes them comfortable for the players (Fox et al., 2017). In addition, IMUs provide objective data available immediately after a training session or game, thus resulting as an easier and faster tool to collect and process data than TMA (Portes et al., 2020). Due to these advantages, IMUs are one of the main technologies adopted by basketball practitioners and sport scientists (Schelling and Torres, 2016; Pino-Ortega et al., 2019; Vázquez-Guerrero et al., 2019a,b; García et al., 2020; Portes et al., 2020; Ransdell et al., 2020). However, IMUs are much more expensive than TMA, which limits their applicability in several contexts, such as non-elite practice (Fox et al., 2017). Additionally, there may be an underestimation when quantifying the external load associated with static activities (e.g., screens, picks, boxing out, low-post situations), as these actions could result in low inertial acceleration loads although encompassing an high muscle activity (Schelling and Torres, 2016). Finally, the use of IMUs during match-play is forbidden in some competitive leagues, limiting the possibility of data collection and comparison (Schelling and Torres, 2016).

While providing descriptive information about external demands in players, TMA cannot quantify the load associated with single activities (Bailey et al., 2017). On the other hand, IMUs cannot systematically detect different activity patterns, such as sprinting versus high-intensity-specific movements (HSM). The use of an integrated approach could provide experts with a quantitative description of the external load associated with single basketball-specific activities. To the best of our knowledge, no previous investigations in basketball have integrated measures of external load collected through TMA with those from IMUs to establish the accumulated load associated with single activities. In the only study conducted in a similar fashion, TMA and IMUs datasets were aligned to calculate the PL associated with single activities in elite netball players, with off-ball guarding and passing corresponding to the highest and lowest PL, respectively (Bailey et al., 2017). A similar approach in basketball may provide relevant insight for sport scientists and coaches regarding load management when planning training sessions. Therefore, the aim of this study was to quantify the external load measured *via* IMUs of high-intensity activities recorded using TMA during official basketball games with respect to different: (1) activity patterns, (2) playing positions, and (3) activities performed with or without ball.

## Materials and Methods

### Participants

Data were collected from 11 adult, male basketball players (age: 20.5 ± 1.1 years, stature: 191.5 ± 8.7 cm, body mass: 86.5 ± 11.3 kg; experience: 8.5 ± 2.4 years) belonging to the same team competing in the Lithuanian third division [Regionu Krepšinio Lyga (RKL)] and the Lithuanian Student League [Lietuvos Studentu Krepšinio Lyga (LSKL)]. Players were grouped according to playing positions including backcourt (*n* = 6, age: 20.9 ± 1.2 years, stature: 185.8 ± 5.5 cm, body mass: 81.0 ± 4.0 kg; experience: 9.2 ± 2.9 years) and frontcourt (*n* = 5, age: 20.1 ± 1.0 years, stature: 198.4 ± 6.5 cm, body mass: 93.0 ± 14.1 kg; experience: 7.6 ± 1.3 years). The inclusion criteria included being part of the team during the entire study period, being free of injuries, completing the standard training program of the team during the entire data collection period and taking part in at least two of the three investigated games. Throughout the study period, players attended 3–4 training sessions per week and competed twice a week (i.e., one game for each league). After verbal and written explanation of the experimental design and potential risks and benefits of the study, written informed consent was gathered from all players. The study design and procedures were approved by the University of Milan Ethics Committee (code: 124/20) and followed the ethical principles for medical research involving human subjects set by the World Medical Association Declaration of Helsinki.

### Design

This observational study was conducted during the in-season period assessing three consecutive home games of the 2019/20 RKL season played between the 7th and 21st of February 2020. According to the FIBA rules, games consisted of four 10-min quarters, with 24-s shot clock, 2-min inter-quarter breaks and a 15-min half-time break (FIBA, 2018). The three games resulted in three losses, with the final scores being 70–85, 78–98, and 80–89, respectively. Each investigated game was recorded and successively analyzed using video-based TMA technique to assess high-intensity activities patterns and their duration. Moreover, the external load measured *via* IMUs (PL and PL/min) was quantified for each player. Successively, TMA and accelerometer datasets were aligned to establish the duration, PL and PL/min of each high-intensity activity instance. Specifically, 32 individual TMAs were conducted across the study, with one player participating in two out of the three investigated games, resulting in a total of 465 sprints, 896 HSMs, and 496 jumps. Each high-intensity activity was assessed according to playing position (backcourt and frontcourt), activity pattern (sprint, HSM and jump), and activities performed with or without ball.

## Procedure

### Time-Motion Analysis

Each of the three games was recorded using a smartphone-embedded camera sampling at 30 Hz (Redmi 5 Plus, Xiaomi, Beijing, China), appropriately positioned to allow a full view of the court. A freely downloadable Android software (Timestamp Camera Free, Bian Di, Google Play Store) that allows to include an on-screen timeline indicating the exact actual time of day (hours, minutes, seconds, and milliseconds) in which activities are happening while recording was used. The recorded files were then exported to a personal computer and a manual TMA was performed using a freely available frame-by-frame software (PotPlayer, Kakao Corporation, South Korea). As previously described (McInnes et al., 1995; Conte et al., 2015; Ferioli et al., 2020c), high-intensity activities were classified into the following three patterns: (1) sprint, identified as forward or backwards activity at a high intensity, characterized by effort and purpose at or close to maximum; (2) high-intensity-specific movements (HSM), which are activities differing from ordinary walking or running performed at high intensity with urgency such as shuffling, rolling, reversing, screening, and cross-over running activities (Conte et al., 2015) and (3) jump, indicated as the time from the initiation of the jumping action to the completion of landing. For each activity, the specified characteristics included the starting and ending time of day (hh:mm:ss.ms), activity pattern (sprint, HSM, or jump), duration (s) and whether activities were performed with or without ball. All activities were examined during live time (i.e., when players are on court and game clock is running). The analysis was carried out by a single experienced video analyst. Intra-tester reliability was determined by having the observer analyze the relative frequency (n/min) and duration (s) of activities during the first quarter of the first game for all players on two separate occasions. The resulting values for the intraclass correlation coefficient and the coefficient of variation (CV) were deemed acceptable (Table 1).

**Table 1:** Intratester reliability of time-motion analysis variables.

	**ICC (90% CI)**	**CV (90% CI)**
***Frequency (n/min)***		
Sprint	0.98 (0.93–0.99)	11.94 (8.71–19.65)
HSM	0.98 (0.95–0.99)	7.29 (5.32–11.99)
Jump	1.00 (1.00–1.00)	0.00 (0.00–0.00)
***Duration (s)***		
Sprint	0.99 (0.97–1.00)	5.49 (4.00–9.03)
HSM	0.88 (0.67–0.96)	11.16 (8.14–18.36)
Jump	1.00 (0.99–1.00)	1.83 (1.33–3.01)

*CI, confidence interval; ICC, intraclass correlation coefficient; CV, coefficient of variation; HSM, high-intensity specific movements*.

### Inertial Movement Analysis

Inertial data for each player were collected using IMUs (Clearsky T6, Catapult Innovation, Melbourne, Australia) firmly positioned in custom-made vests between the players' scapulae. Players were assigned the same device every game to minimize any variations in data between IMUs. PL was calculated *via* Catapult proprietary software (Catapult Openfield, v1.17, Catapults Innovations, Melbourne, Australia) as the sum of the accelerations across all axes during movement using the tri-axial accelerometer component of the IMU (Nicolella et al., 2018). The index takes into account the instantaneous rate of change of acceleration and divides it by a scaling factor of 100 (Nicolella et al., 2018). The reliability of PL (within-device CV = 0.91–1.05%, between-device CV = 1.02–1.90%) has been previously assessed (Boyd et al., 2011) and this metric has been widely used in basketball (Schelling and Torres, 2016; Fox et al., 2017, 2018; Pino-Ortega et al., 2019; Vázquez-Guerrero et al., 2019a,b; Conte et al., 2020; García et al., 2020; Lukonaitienė et al., 2020; Portes et al., 2020; Ransdell et al., 2020; Russell et al., 2020).

### Integration of TMA and Accelerometer Data

The IMU and TMA datasets were aligned using the starting and ending time of each activity coded with the “Timestamp Camera Free” app. Immediately after each game, PL data were downloaded and stored *via* Catapult proprietary software. This procedure allowed the quantification of the PL, PL/min and duration for each coded high-intensity activity. This analysis resulted in a fully comprehensive dataset containing all activities.

### Statistical Analysis

Data are presented as mean ± SD for each dependent variable (i.e., PL, PL/min, duration) and analyzed using linear mixed models, which correctly deals with missing values and repeated measures. Separate models were used (one for each dependent variable) to assess the differences between the three investigated activity patterns (i.e., sprint, HSM, and jump). In these models, activity pattern was considered the fixed effect, while player, game and quarter were used as random effects. In case of statistically significant differences, Tukey *post hoc* analyses were run. Additionally, linear mixed models were also used to assess the differences between playing positions (backcourt and frontcourt) for each dependent variable during each activity. In these models, playing position was used as fixed effect and player, game and quarter as random effects. Finally, linear mixed models were also used to assess the differences between activities performed with and without ball (fixed effect) for each investigated dependent variable with playing position, player, game and quarter as random effect. Significance was set at *p* < 0.05. Linear mixed models and *post-hoc* analyses were conducted using the “lmerTest” and “emmeans” packages, respectively, in RStudio (R.3.5.2, R Foundation for Statistical Computing). The magnitude of differences for pairwise comparisons was assessed using effect size (ES) with 95% confidence intervals. ESs were calculated using JASP team 2019 (v0.10.2) and interpreted as <0.2 = *trivial*, 0.20–0.59 = *small*, 0.60–1.19 = *moderate*, 1.2–1.99 = *large*, and ≥2.0 = *very large* (Hopkins et al., 2009).

## Results

Table 2 shows activity frequency and the mean and standard deviation for all variables for each activity pattern. The linear mixed model analysis highlighted significant differences in PL (*p* < 0.001), PL/min (*p* = 0.025) and duration (*p* < 0.001) among activity patterns. First, *post-hoc* analysis showed higher PL values for sprints compared with jumps (*p* < 0.001, ES = 0.68, *moderate*) and for HSMs compared with jumps (*p* < 0.001, ES = 0.58, *small*). No significant differences in PL were found between sprints and HSMs (*p* = 0.960, ES = 0.02, *trivial*). Second, higher PL/min values were evident for sprints compared with jumps (*p* = 0.023, ES = 0.22, *small*). No significant differences in PL/min were found in sprints compared with HSMs (*p* = 0.100, ES = 0.16, *trivial*) and in HSMs compared with jumps (*p* = 0.592, ES = 0.07, *trivial*). Finally, higher duration values were reported in HSMs compared with sprints (*p* = 0.002, ES = 0.17, *trivial*) and jumps (*p* < 0.001, ES = 0.81, *moderate*), and in sprints compared with jumps (*p* < 0.001, ES = 1.10, *moderate*).

**Table 2:** Activity frequency and mean ± standard deviation for all variables in each activity type.

	**Sprint** ** (*N* = 465)**	**HSM** ** (*N* = 896)**	**Jump** ** (*N* = 496)**
PL (AU)	0.44 ± 0.43^*^	0.44 ± 0.44^#^	0.22 ± 0.16
PL/min (AU/min)	21.69 ± 14.05^†^	19.61 ± 13.02	18.70 ± 13.43
Duration (s)	1.18 ± 0.60^‡^^¥^	1.32 ± 0.93^¢^	0.72 ± 0.11

*PL, PlayerLoad^TM^; PL/min, PlayerLoad^TM^ per minute; AU, arbitrary units*.

* *Significant difference with jump [p < 0.001; mean difference = 0.22 (95% CI: 0.18–0.26); ES = 0.68, moderate (95% CI 0.55–0.81)]*;

# *significant difference with jump [p < 0.001; mean difference = 0.21 (95% CI: 0.17–0.25); ES = 0.58, small (95% CI: 0.47–0.69)]*;

† *significant difference with jump [p = 0.023; mean difference = 2.99 (95% CI: 1.25–4.73); ES = 0.22, small (95% CI: 0.09–0.34)]*;

‡ *Significant difference with HSM [p = 0.002; mean difference = -0.14 (95% CI:-0.23– -0.05); ES = -0.17, trivial (95% CI: -0.28– -0.06)]*;

¥ *significant difference with jump: [p < 0.001; mean difference = 0.46 (95% CI: 0.41–0.52); ES = 1.10, moderate (95% CI: 0.96–1.23)]*;

¢ *significant difference with jump [p < 0.001; mean difference = 0.60 (95% CI: 0.52–0.69); ES = 0.81, moderate (95% CI: 0.69–0.92)]*.

Differences in PL, PL/min and duration for all activity patterns between playing positions are displayed in Table 3. Significantly higher values were found for jumps duration in backcourt compared with frontcourt players (*p* < 0.001, ES = 0.33, *small*). No other significant differences were found between playing positions for all the other variables.

**Table 3:** Differences in PL, PL/min and duration for all activity patterns between backcourt and frontcourt positions.

						**95% CI for MD**			**95% CI for ES**		
**Dependent variables**	**Backcourt**	**Frontcourt**	**% difference**	***P* value**	**MD**	**Lower**	**Upper**	**ES**	**Lower**	**Upper**	**Interpretation**
***Sprint (BC=313; FC=152)***											
PL	0.45 ± 0.46	0.43 ± 0.38	4.65	0.920	0.02	−0.06	0.11	0.05	−0.14	0.25	*Trivial*
PL/min	22.15 ± 15.07	20.74 ± 11.66	6.80	0.909	1.40	−1.33	4.13	0.10	−0.09	0.30	*Trivial*
Duration	1.16 ± 0.58	1.22 ± 0.62	4.92	0.579	−0.06	−0.18	0.06	−0.10	−0.30	0.09	*Trivial*
***HSM (BC=534; FC=362)***											
PL	0.45 ± 0.48	0.41 ± 0.39	9.76	0.783	0.04	−0.02	0.10	0.09	−0.04	0.23	*Trivial*
PL/min	19.49 ± 13.50	19.78 ± 12.31	1.47	0.550	−0.28	−2.02	1.46	−0.02	−0.16	0.11	*Trivial*
Duration	1.37 ± 0.99	1.25 ± 0.83	9.60	0.196	0.13	0.00	0.25	0.14	0.00	0.27	*Trivial*
***Jump (BC=286; FC=210)***											
PL	0.23 ± 0.17	0.22 ± 0.15	4.55	0.872	0.01	−0.02	0.04	0.03	−0.14	0.21	*Trivial*
PL/min	18.41 ± 13.84	19.09 ± 12.86	3.56	0.569	−0.68	−3.08	1.72	−0.05	−0.23	0.13	*Trivial*
Duration	0.73 ± 0.10	0.70 ± 0.12	4.29	** <0.001**	0.04	0.02	0.05	0.33	0.15	0.51	*Small*

*Data presented as mean ± standard deviation*.

*BC, activity frequency for backcourt; FC, activity frequency for frontcourt; PL, PlayerLoad^TM^; PL/min, PlayerLoad^TM^ per minute; HSM, high–intensity specific movements; MD, mean difference; CI, confidence interval; ES, effect size. Bolded P value represents significant difference (P < 0.05)*.

Differences in PL, PL/min and duration between activities with and without ball are displayed in Table 4. When considering all activity patterns combined, significantly higher values were found for PL (*p* < 0.001, ES = 0.28, *small*) and duration (*p* < 0.001, ES = 0.43, *small*) when players did not have the ball. With respect to HSMs, PL/min was significantly higher with ball (*p* = 0.036, ES = 0.14, *trivial*), while duration was longer without ball (*p* < 0.001, ES = 0.34, *small*). No other significant differences were found for all the other variables.

**Table 4:** Differences in PL, PL/min and duration for activities with and without ball.

					**95% CI for MD**			**95% CI for ES**		
**Dependent variables**	**No ball**	**Ball**	***P* value**	**MD**	**Lower**	**Upper**	**ES**	**Lower**	**Upper**	**Interpretation**
***All activities (NB=1,230, B=628)***										
PL (AU)	0.42 ± 0.43	0.31 ± 0.31	** <0.001**	0.11	0.07	0.15	0.28	0.18	0.38	*Small*
PL/min (AU/min)	19.96 ± 13.45	19.75 ± 13.41	0.730	0.21	−1.08	1.50	0.02	−0.08	0.11	*Trivial*
Duration (s)	1.23 ± 0.84	0.91 ± 0.49	** <0.001**	0.32	0.25	0.39	0.43	0.33	0.53	*Small*
***Sprint (NB=321, B=144)***										
PL (AU)	0.46 ± 0.45	0.42 ± 0.38	0.445	0.04	−0.04	0.13	0.10	−0.10	0.29	*Trivial*
PL/min (AU/min)	21.45 ± 13.57	22.22 ± 15.10	0.195	−0.78	−3.55	2.00	−0.06	−0.25	0.14	*Trivial*
Duration (s)	1.21 ± 0.64	1.11 ± 0.47	0.050	0.10	−0.01	0.22	0.17	−0.02	0.37	*Trivial*
***HSM (NB=715, B=181)***										
PL (AU)	0.45 ± 0.44	0.38 ± 0.39	0.237	0.07	−0.01	0.14	0.15	−0.01	0.32	*Trivial*
PL/min (AU/min)	19.24 ± 12.94	21.04 ± 13.28	**0.036**	−1.80	−3.92	0.33	−0.14	−0.30	0.03	*Trivial*
Duration (s)	1.39 ± 0.96	1.07 ± 0.73	** <0.001**	0.32	0.17	0.47	0.34	0.18	0.51	*Small*
***Jump (NB=194, B=302)***										
PL (AU)	0.24 ± 0.19	0.21 ± 0.15	0.129	0.02	0.00	0.05	0.15	−0.03	0.33	*Trivial*
PL/min (AU/min)	20.11 ± 14.87	17.79 ± 12.35	0.075	2.32	−0.10	4.74	0.17	−0.01	0.35	*Trivial*
Duration (s)	0.71 ± 0.12	0.73 ± 0.10	0.072	−0.02	−0.04	0.00	−0.15	−0.33	0.03	*Trivial*

*Data presented as mean ± standard deviation*.

*NB, activity frequency without ball; B, activity frequency with ball; PL, PlayerLoad; PL/min, PlayerLoad per minute; HSM, high-intensity specific movements; MD, mean difference; CI, confidence interval; ES, effect size. Bolded P value represents significant difference (P < 0.05)*.

## Discussion

The present study is the first to quantify the external load measured *via* IMUs during single high-intensity activities recorded using TMA in basketball games. This is also the first study to compare the external load quantified using IMUs during single high-intensity activities according to playing positions and activities performed with or without ball. The main results revealed PL values were statistically lower in jumps compared to sprints (ES, *moderate*) and HSMs (ES, *small*), while PL/min was statistically lower in jumps compared to sprints (ES, *small*). With respect to playing positions, jumps duration was statistically longer in backcourt compared with frontcourt players (ES, *small*). Finally, PL and duration values appeared to be higher in all activity patterns combined when players did not have the ball with a *small* effect size.

Although multiple investigations have measured the PL and PL/min during official games and games-based drills in basketball (Schelling and Torres, 2016; Fox et al., 2018; Vázquez-Guerrero et al., 2018, 2019a,b; Pino-Ortega et al., 2019; Conte et al., 2020; Fernández-Leo et al., 2020; García et al., 2020; O'Grady et al., 2020; Portes et al., 2020; Ransdell et al., 2020), no study has assessed the external load imposed during single basketball-specific activities measured *via* TMA. To the best of our knowledge, a similar approach was previously used only in netball, with two official games analyzed *via* TMA and IMUs, revealing that on average the highest PL was registered during off-ball guarding actions (Bailey et al., 2017). Considering the importance of load monitoring for the optimization of the training process (Conte et al., 2018), quantifying the load imposed by high-intensity basketball activities can help practitioners in prescribing an adequate training load. Accordingly, the ability to perform at higher intensities has been reported to be crucial to compete at a higher level, with elite basketball players displaying a better ability to sustain high-intensity efforts (Ferioli et al., 2018) and elite basketball games involving more and longer high-intensity activities (Ferioli et al., 2020c) than lower level competitions. The findings of the present study show that PL, which is one of the main measures adopted to assess training volume in basketball (Russell et al., 2020), was higher in sprints and HSMs compared to jumps. Differently, when considering PL/min, which refers to the training intensity, the only significant difference (ES, *small*) was found in sprints compared to jumps, while no statistically significant differences were evident for HSM compared to the other activity patterns. This discrepancy in the results between sprint and HSM when considering training volume and intensity could be explained by the longer (*p* = 0.002) duration of HSMs compared to sprints, that would produce a similar volume and a lower intensity. However, caution is advised when interpreting such results, as differences in duration between sprints and HSMs were *trivial*. These findings should be considered when planning training sessions on a regular basis, as using different proportions of each activity pattern is likely to produce different total load during sessions with the same duration.

It has been shown that playing position can affect players' physical demands during official games (Puente et al., 2017; Stojanović et al., 2018; Vázquez-Guerrero et al., 2018, 2019b; Pino-Ortega et al., 2019; Ferioli et al., 2020b; Fernández-Leo et al., 2020; García et al., 2020). Specifically, while previous research has identified positional differences in the frequency and duration of high-intensity activities during basketball games (Stojanović et al., 2018), there is no indication of the external load registered during single high-intensity activities according to playing position. The findings indicated that, although backcourt players performed a higher number of high-intensity activities compared to frontcourt players (backcourt: sprint, *n* = 313; HSM, *n* = 534; jumps, *n* = 286; *N* = 1,133; frontcourt: sprint, *n* = 152; HSM, *n* = 362; jumps, *n* = 210; *N* = 724), *trivial-to-small* differences were evident in PL, PL/min and duration between backcourt and frontcourt players during each high-intensity activity. Therefore, basketball coaches and practitioners should consider designing drills with a different number of high-intensity activities between backcourt and frontcourt players although with a similar volume, intensity and duration. Regarding average high-intensity activity duration, the only significant difference was a higher (ES, *small*) value for jumps in backcourt compared with frontcourt players. This may be due to the different physical characteristics observed between playing positions (Ferioli et al., 2018), as backcourt players are usually smaller and lighter and therefore able to jump for a longer time compared to frontcourt players, who are taller and heavier (Schelling and Torres, 2016; García et al., 2020).

Previous investigations described the frequency and duration of activities performed with ball during official basketball games (Scanlan et al., 2011, 2012, 2015; Ferioli et al., 2020b). Specifically, three previous studies (Scanlan et al., 2011, 2012, 2015) evaluated the duration of dribbling activities in basketball players. In addition, Ferioli et al. (2020b) assessed positional differences during activities performed with ball, showing that guards, forwards and centers spent 11.9 ± 5.9, 3.5 ± 1.3, and 2.9 ± 1.1% of live playing time in possession of the ball, of which 19.0 ± 13.2, 35.2 ± 16.0, and 36.7 ± 11.4% engaged in high-intensity activities, respectively. However, this study did not explore differences between activities performed with and without ball (Ferioli et al., 2020b). The present study is the first to explore differences in PL, PL/min and duration between high-intensity activities performed with and without ball, showing that overall activities without ball displayed higher PL and duration with *small* effect sizes compared to overall activities with ball. This result suggests that high-intensity activities performed without ball during basketball games elicited higher external load volume due to the longer duration. Differently, high-intensity activities with and without ball produced a *trivial* difference in the external load intensity (PL/min). However, when considering high-intensity activities separately, a statistically higher PL/min and shorter duration were shown in HSMs performed with ball compared to HSMs without ball, even though caution is advised since *trivial-to-small* effect sizes were reported. A likely explanation of these results is that such activities are usually performed when attacking the basket and/or looking to create a scoring opportunity (e.g., dribble crossovers, post-up spin moves), thus producing a higher acceleration load. Therefore, quickness of executions seems to be crucial when performing these activities and may translate to better performance and team success.

There are some limitations of the present study that must be acknowledged. First, the recruited basketball players in this study were competing in the same male basketball league, and therefore the findings might not be generalizable to basketball players competing in other male or female competitions, calling for further studies on these specific basketball populations. Second, this study examined activities performed with and without ball without accounting for positional differences, as guards, forwards and centers have been shown to spend different proportions of live time in possession of the ball (Ferioli et al., 2020b). Finally, the current investigation focused on quantifying the external load associated only with high-intensity activities, as they are the most crucial for team success during a competitive game. However, further research also describing low- and moderate-intensity activities could prove useful information about all activity patterns to better describe the external load in basketball games.

### Practical Applications

From a practical standpoint, the findings of the present study offer information that can assist practitioners during training planning. Firstly, the higher PL detected during sprints and HSMs compared to jumps indicates that the selection of different types of drills including different proportions of high-intensity activity patterns may influence the total load of the session. Secondly, despite backcourt players performing a higher number of high-intensity activities compared to frontcourts, the external load for each investigated high-intensity activity was similar between positions. Therefore, the use of training drills encompassing activities of similar volume, intensity and duration between positions seems justified. Moreover, a greater use of drills including a higher number of high-intensity activities performed without ball may result in higher total volume. Considering intensity, basketball coaches are suggested to design training drills with high-intensity activities with and without ball with a similar PL/min (~20 AU/min). Furthermore, focusing on quickness of execution of HSMs performed with ball may be paramount since such activities have shown shorter duration and higher intensity compared to HSMs without ball, and are often employed when looking to create scoring opportunities.

## Conclusion

The present study reports novel findings regarding the external load sustained when performing high-intensity activities during official basketball games. Different high-intensity activity patterns performed during basketball games are characterized by different levels of external load, with a lower PL in jumps compared to sprints and HSMs, and a lower PL/min in jumps compared to sprints. With respect to playing positions, jumps duration is longer in backcourt compared with frontcourt players. Finally, PL and duration values of all high-intensity movement patterns appear to be higher when players are not in possession of the ball. The findings of the present study should be considered by coaches and sport scientists for a better development of training sessions involving various types of high-intensity activities, as external load may be affected by their development in accordance with various factors (i.e., playing positions and activity performed with or without ball).

## Data Availability Statement

The raw data supporting the conclusions of this article will be made available by the authors, without undue reservation.

## Ethics Statement

The studies involving human participants were reviewed and approved by University of Milan Ethics Committee. The patients/participants provided their written informed consent to participate in this study.

## Author Contributions

MP: data collection, time-motion analysis, writing of the original manuscript draft, interpretation of results, visualization, contribution to conceptualization, and design. DF: conceptualization and design, supervision, interpretation of results, reviewing, and editing. RB: resourcing, data collection, reviewing, and editing. AL: funding acquisition, submission of the protocol to the ethics committee, supervision, reviewing, and editing. DC: conceptualization and design, resourcing, supervision, contribution to data collection, statistical analysis, interpretation of results, reviewing, and editing. All authors have read and agreed to the published version of the manuscript.

## Conflict of Interest

DF was employed by company CS Pallacanestro Trapani SSDARL. The remaining authors declare that the research was conducted in the absence of any commercial or financial relationships that could be construed as a potential conflict of interest.
